# Recombinant Human Parathyroid Hormone Effect on Health-Related Quality of Life in Adults With Chronic Hypoparathyroidism

**DOI:** 10.1210/jc.2017-01471

**Published:** 2017-11-01

**Authors:** Tamara J Vokes, Michael Mannstadt, Michael A Levine, Bart L Clarke, Peter Lakatos, Kristina Chen, Rebecca Piccolo, Alan Krasner, Dolores M Shoback, John P Bilezikian

**Affiliations:** 1 Section of Endocrinology, University of Chicago Medicine, Chicago, Illinois; 2 Endocrine Unit, Massachusetts General Hospital and Harvard Medical School, Boston, Massachusetts; 3 Division of Endocrinology and Diabetes and Center for Bone Health, Children’s Hospital of Philadelphia, Philadelphia, Pennsylvania; 4 Mayo Clinic Division of Endocrinology, Diabetes, Metabolism, and Nutrition, Rochester, Minnesota; 5 1st Department of Medicine, Semmelweis University, Budapest, Hungary; 6 Shire Human Genetic Therapies, Inc., Lexington, Massachusetts; 7 Endocrine Research Unit, SF Department of Veterans Affairs Medical Center, University of California, San Francisco, California; 8 Division of Endocrinology, College of Physicians and Surgeons, Columbia University, New York, New York

## Abstract

**Context:**

Reduced health-related quality of life (HRQoL) is common in patients with hypoparathyroidism treated conventionally with calcium and active vitamin D supplements.

**Objective:**

To examine the effects of recombinant human parathyroid hormone [rhPTH(1-84)] on HRQoL as measured by the 36-Item Short-Form Health Survey (SF-36) during a multinational, randomized, placebo-controlled study.

**Patients:**

Adults (N = 122) with chronic hypoparathyroidism.

**Intervention(s):**

After an optimization period when calcium and/or active vitamin D supplements were adjusted to reach target serum calcium levels (8.0 to 9.0 mg/dL; 2.0 to 2.2 mmol/L), patients were randomly assigned to receive placebo (n = 39) or rhPTH(1-84) (n = 83) (starting dose, 50 μg/d, could be titrated up to 100 μg/d); supplement doses were adjusted to maintain target serum calcium levels.

**Main Outcome Measure(s):**

Change from baseline (postoptimization, at randomization) to week 24 in HRQoL as assessed by the SF-36.

**Results:**

Overall, the between-group differences were not statistically significant. However, in the rhPTH(1-84) group, there were significant improvements in the physical component summary score (*P* = 0.004), and in body pain (*P* < 0.05), general health (*P* < 0.05), and vitality (*P* < 0.001) domains as compared with baseline values. In the placebo group, there were no significant changes for any domains. The magnitude of change between 0 and 24 weeks in SF-36 scores was negatively correlated with baseline scores, such that patients with lower HRQoL at baseline were more likely to experience improvement in response to treatment.

**Conclusion:**

Treatment with rhPTH(1-84) may improve HRQoL in adults with hypoparathyroidism.

## Teaser

In a double-blind, 6-month trial, patients receiving rhPTH(1-84) had improvements in some SF-36 domains but not those receiving placebo; differences between groups were not statistically significant.

Hypoparathyroidism is a rare endocrine disorder characterized by hypocalcemia and hyperphosphatemia due to low or absent circulating levels of parathyroid hormone (PTH). Conventional treatment of hypoparathyroidism consists of oral calcium supplements and active forms of vitamin D. Conventional treatment is often associated with incomplete control of symptoms and poor well-being (1, 2), suggesting that there is reduced health-related quality of life (HRQoL) in this patient population (3). Several recent studies have reported various aspects of well-being that were impaired in patients with hypoparathyroidism compared with healthy individuals or patients with other metabolic disorders. A study of German patients reported that 25 women with postsurgical hypoparathyroidism had higher global complaint scores, with predominant increases in subscores for anxiety, compared with women who had prior thyroid surgery but retained normal parathyroid function (4). Similarly, another study found that Danish women with both postsurgical hypothyroidism and hypoparathyroidism had lower scores in most domains of the 36-Item Short-Form Health Survey (SF-36) than women with only postsurgical hypothyroidism or than age-matched control subjects (5). In a Norwegian study of patients with hypoparathyroidism, SF-36 scores were significantly lower than the normative data, with lower scores observed in surgical cases compared with nonsurgical cases (6). A study of 688 patients from a Danish national registry who had postsurgical hypoparathyroidism showed these patients had a higher incidence of depression and other psychiatric symptoms compared with 2064 matched control subjects (7). Finally, a US, Internet-based survey of 374 patients with hypoparathyroidism showed that most had fatigue, as well as emotional and cognitive impairments (8). It is also noteworthy that these symptoms are often unexpected or underappreciated. For example, in a US study, 340 patients with postsurgical hypoparathyroidism had symptoms that were considerably worse than anticipated by 102 experienced endocrine surgeons and 200 healthy subjects given the routine preoperative description of hypoparathyroidism as a complication of neck surgery (9). Finally, all prior studies of PTH replacement therapy in hypoparathyroidism have shown that HRQoL at baseline (*i.e.,* while receiving conventional therapy) was lower than in the general population (10–13).

In this study, we analyzed data from a randomized, double-blind, placebo-controlled, phase 3 study, REPLACE (14, 15), the clinical trial that led to the approval of recombinant human PTH [rhPTH(1-84)] for the treatment of chronic hypoparathyroidism in adults (16, 17) to determine whether rhPTH(1-84) could improve HRQoL over what can be achieved with optimized conventional treatment with calcium and active vitamin D alone. This information would add to previous studies that have not been conclusive or lacked a placebo-treated control group (10–13). Additionally, as a multinational study, REPLACE presented an opportunity to conduct a post hoc analysis with regard to geographic heterogeneity among the three regions that participated in the study: North America, Western Europe, and Central/Eastern Europe, which only included Hungary.

## Materials and Methods

### Patients and study design

Detailed inclusion and exclusion criteria have been reported by Mannstadt *et al.* (15) and Clarke *et al.* (14). Briefly, the study enrolled patients between 18 and 85 years old with chronic hypoparathyroidism based on hypocalcemia and documented serum PTH levels below the lower limit of the normal range. Patients with known or suspected mutations in the calcium-sensing receptor gene were excluded. The study was conducted in accordance with Good Clinical Practice guidelines and the Declaration of Helsinki, the protocol was approved by the institutional review boards, and all study participants provided written informed consent.

After enrollment, patients underwent an optimization period lasting 2 to 16 weeks, during which active vitamin D (calcitriol or alfacalcidol) and calcium doses were adjusted to achieve albumin-corrected serum calcium levels between 7.5 mg/dL (1.9 mmol/L) and the upper limit of normal, but ideally within a target range of 8.0 to 9.0 mg/dL (2.0 to 2.2 mmol/L). Serum levels of 25-hydroxyvitamin D were optimized to be between 30 ng/mL (75 nmol/L) and 100 ng/mL (250 nmol/L), and magnesium deficiency [defined as values <1.3 mEq/L (0.7 mmol/L)] was corrected.

After daily doses of calcium and active vitamin D were stable for 2 weeks, patients were randomly assigned in a double-blinded manner to receive once-daily subcutaneous injections of either placebo or 50 μg rhPTH(1-84) in a 1:2 ratio. The 24-week treatment period began with a titration phase, during which the rhPTH(1-84) dose was increased and active vitamin D and calcium doses were reduced while maintaining albumin-corrected serum calcium at or above baseline levels [target level: between 7.5 mg/dL (1.9 mmol/L) and the upper limit of normal]. The first increase of rhPTH(1-84) to 75 μg/d could occur at week 2, and the second increase to 100 μg/d could occur at week 4. The titration phase was followed by a maintenance phase for the remainder of the treatment period. The dose of rhPTH(1-84) could be decreased at any time, if necessary, but not increased during this latter period. At any time during the treatment period, adjustments were permitted in the doses of active vitamin D and calcium supplements to maintain serum calcium in the target range and to reduce hypercalciuria. After 24 weeks, rhPTH(1-84) was discontinued, baseline oral calcium and active vitamin D were resumed, and patients were monitored for another 4 weeks.

### HRQoL assessments and statistical analysis

The impact of rhPTH(1-84) on HRQoL was assessed in REPLACE using the SF-36, version 2, as an exploratory end point. The questionnaires were administered at randomization (postoptimization baseline) and after 4, 12, and 24 weeks of study drug treatment. The SF-36 survey consists of 36 questions grouped into eight domains of physical and mental health: physical functioning (PF), role-physical (RP), bodily pain (BP), general health (GH), vitality (VT), social functioning (SF), role-emotional, and mental health (MH). These eight domains are then summarized into a physical component summary (PCS) score and an MH component summary (MCS) score. There is a limited range of responses for the survey questions, which are scored using the Likert method of summated ratings. Because the questions differed in the maximum possible score on the scale, each score was recalculated on a 0 to 100 scale expressed as a norm-based score [*i.e.,* relative to 2009 normal population mean (standard deviation) values of 50 (10)] (18), with lower scores corresponding to greater disability. The minimally important difference (MID) for group mean for the individual domains of SF-36 version 2 and summary scores are between 2 and 3 points (18). These MID values were derived from anchors that are relevant for all patients regardless of the specific condition (18).

This analysis used data based on the intent-to-treat population that had at least one postbaseline HRQoL measurement (14). Within- and between-treatment–group analyses of HRQoL overall and by geographic region were prespecified. However, the methods used to analyze this exploratory end point were updated from the initial statistical analysis plan to use a mixed-effect model for repeated measures (MMRM) analysis. The MMRM analysis has the advantage of assessing data from all visits simultaneously, using a restricted maximum likelihood–based approach (19). The model included the fixed categorical effects of rhPTH(1-84) treatment, region, sex, visit, and treatment-by-visit interaction, as well as the continuous, fixed covariates of age, baseline HRQoL, and baseline-by-visit interaction. Within-patient errors were modeled using an unstructured covariance matrix. All analyses were performed using SAS version 9.3 (SAS Institute, Cary, NC).

## Results

Of the 122 patients with at least one postbaseline HRQoL measurement, 83 were in the rhPTH(1-84) group and 39 were in the placebo group, reflecting the 2:1 randomization. Table 1 summarizes key patient demographic and clinical characteristics at baseline (*i.e.,* postoptimization randomization). Similar to the demographics for hypoparathyroidism in the United States and elsewhere (2), most patients were women (rhPTH[1-84], 77%; placebo, 82%) who developed hypoparathyroidism after thyroid surgery (rhPTH[1-84], 71%; placebo, 72%). There was a wide range of daily doses of calcium (1.0 to 12.0 g/d) and active vitamin D (calcitriol or equivalent dose of 0.3 to 4.0 µg/d), but there was no statistically significant difference between treatment groups at baseline. Likewise, there were no statistically significant differences at baseline between treatment groups regarding age, sex, body mass index, duration of hypoparathyroidism, etiology, albumin-corrected serum calcium level, PTH levels, and SF-36 PCS and MCS. At baseline, several SF-36 domain scores were below those of the norm-based population, although the differences were small [rhPTH(1-84) and placebo, respectively, mean (standard error): GH, 46.1 (1.3) and 46.3 (2.0); RP, 47.8 (1.0) and 46.3 (1.6); BP, 48.3 (1.2) and 48.1 (1.4); SF, 48.8 (1.2) and 48.1 (1.6); PF, 49.2 (1.0) and 45.4 (1.5)].

**Table 1.** Patients’ Baseline Characteristics

Variable	Placebo (n = 39)	rhPTH(1-84) (n = 83)	*P* Value*^a^*
Age, mean (SD), y	49.5 (13.3)	46.6 (12.3)	
Female	32 (82.1)	64 (77.1)	0.639
Body mass index, mean (SD), kg/m^2^	28.9 (5.4)	29.1 (6.1)	
Duration of hypoparathyroidism, y			
Mean (SD)	11.8 (8.1)	14.6 (11.2)	
≤5	9 (23.1)	15 (18.1)	
>5–10	13 (33.3)	27 (32.5)	
>10	17 (43.6)	41 (49.4)	
Postsurgical etiology	28 (71.8)	59 (71.1)	1.000
Prescribed calcium dose at baseline, mg/d			
Mean (SD)	1932 (892.0)	2185 (1477.3)	
0−2000	29 (74.4)	56 (67.5)	
>2000	10 (25.6)	27 (32.5)	
Prescribed vitamin D dose at baseline, µg/d*^b^*			
Mean (SD)	1.1 (0.7)	1.1 (0.9)	
Low dose	3 (7.7)	6 (7.2)	
Medium dose	12 (30.8)	21 (25.3)	
High dose	24 (61.5)	56 (67.5)	
Albumin-corrected serum calcium at baseline, mean (SD), mg/dL	8.6 (0.6)	8.5 (0.8)	
Parathyroid hormone at baseline, mean (SD), ng/L*^c^*	6.5 (6.5)	6.3 (8.7)	

Data given as no. (%) unless otherwise indicated.

^a^ Calculated based on *t* tests, except for the variables of female sex and etiology, which were based on the Fisher exact test.

^b^ For calcitriol, low dose is ≤0.25 µg/d; medium dose, >0.25−0.5 µg/d; and high dose, >0.5 µg/d. For alfacalcidol, low dose is <0.50 µg/d; medium dose, >0.5−1.0 µg/d; and high dose, is >1.0 µg/d.

^c^ Normal range in adults is 14−72 ng/L.

The multinational REPLACE study enrolled patients at sites in Western Europe (Belgium, Denmark, France, the United Kingdom, and Italy), Central/Eastern Europe (only Hungary), and North America (Canada and the United States). Baseline SF-36 scores within each region are listed in Table 2 for each treatment arm; there was a range of scores across the regions for any one SF-36 domain, but there were no statistically significant differences between treatment groups in any given region except Hungary. The baseline SF-36 scores tended to be higher in the patients randomly assigned to the rhPTH(1-84) arm than in those in the placebo arm, with between-group differences significant for the PF (*P* = 0.031) and VT (*P* = 0.025) domains and approaching significance for RP (*P* = 0.073).

**Table 2.** Regional Baseline SF-36 Scores

SF-36 Domain	Hungary (n = 23)			North America (n = 62)			Western Europe (n = 37)		
	Placebo, n = 7	rhPTH(1-84) (n = 16)	*P* Value	Placebo (n = 20)	rhPTH(1-84) (n = 42)	*P* Value	Placebo (n = 12)	rhPTH(1-84) (n = 25)	*P* Value
PF	42.1 (1.7)	49.4 (1.9)	0.031	51.6 (1.5)	51.3 (1.3)	0.888	40.0 (3.3)	45.5 (2.1)	0.151
RP	40.8 (2.4)	48.5 (2.5)	0.073	52.2 (1.6)	50.6 (1.1)	0.411	42.6 (3.3)	43.3 (2.1)	0.843
BP	48.1 (4.1)	49.7 (3.0)	0.754	50.8 (1.7)	49.9 (1.5)	0.708	47.0 (3.0)	46.0 (2.2)	0.775
GH	41.1 (3.6)	45.3 (2.7)	0.379	53.9 (1.8)	50.4 (1.5)	0.192	41.0 (3.3)	40.6 (2.4)	0.926
VT	45.0 (4.0)	55.9 (2.5)	0.025	52.0 (1.8)	50.6 (1.6)	0.589	42.4 (4.0)	43.4 (2.5)	0.829
SF	46.6 (4.0)	51.1 (2.1)	0.292	51.1 (1.9)	51.0 (1.4)	0.991	44.0 (3.2)	43.5 (2.3)	0.908
RE	43.2 (4.1)	47.7 (3.0)	0.409	50.4 (2.0)	52.9 (1.0)	0.215	45.7 (3.4)	44.6 (2.0)	0.766
MH	46.4 (5.1)	53.3 (2.3)	0.164	52.3 (1.6)	53.1 (1.4)	0.742	46.1 (3.5)	44.8 (2.2)	0.754
PCS	42.5 (1.5)	47.8 (2.2)	0.146	52.1 (1.5)	49.6 (1.4)	0.266	41.5 (3.2)	44.2 (2.1)	0.479
MCS	46.5 (5.1)	52.7 (2.4)	0.216	51.3 (2.1)	52.8 (1.4)	0.533	46.5 (3.5)	44.2 (2.2)	0.575

Data given as mean (SE) unless otherwise indicated. For all domains, normal population has the mean of 50 and a standard deviation of 10. Abbreviation: RE, role-emotional.

Table 3 presents the mean baseline and 24-week values for the eight SF-36 domains and two composite scores (PCS and MCS). For each treatment group, we included the unadjusted means and standard errors; to analyze the effect of treatment, we used the MMRM-adjusted least squares (LS) mean change from baseline with corresponding *P* values comparing within and between treatment groups. Within the rhPTH(1-84) group, four SF-36 domains were statistically significant when analyzing LS mean change from baseline from the multivariate adjusted model [BP, 2.0 (*P* < 0.05); GH, 2.0 (*P* < 0.05);VT, 3.7 (*P* < 0.001); and PCS, 2.0 (*P* < 0.05)]. In contrast, there were no significant changes from baseline in any SF-36 domain within the placebo group. The between-group differences in LS mean change from baseline to 24 weeks were not statistically significant.

**Table 3.** SF-36 Scores at Baseline and After 24 Weeks of Treatment

SF-36 Domain	Placebo (n=39)				rhPTH(1-84) (n=83)				rhPTH(1-84) vs Placebo
	Baseline	Week 24	LS Mean Change (SE)*^a^*	*P* Value^*b*^	Baseline	Week 24	LS Mean Change (SE)*^a^*	*P* Value*^b^*	*P* Value*^c^*
PF	45.4 (1.5)	46.8 (1.7)	1.3 (1.2)	0.278	49.2 (1.0)	50.1 (1.1)	1.4 (0.8)	0.071	0.894
RP	46.3 (1.6)	47.0 (1.6)	−0.9 (1.3)	0.476	47.8 (1.0)	49.7 (1.0)	1.2 (0.9)	0.167	0.143
BP	48.1 (1.4)	47.9 (1.7)	−0.6 (1.4)	0.662	48.3 (1.2)	50.8 (1.3)	2.0 (1.0)	0.042	0.106
GH	46.3 (2.0)	46.6 (2.1)	−0.2 (1.1)	0.843	46.1 (1.3)	48.5 (1.2)	2.0 (0.8)	0.016	0.088
VT	47.7 (1.8)	50.0 (1.9)	1.6 (1.4)	0.258	49.5 (1.3)	53.0 (1.1)	3.7 (1.0)	<0.001	0.173
SF	48.1 (1.6)	48.5 (1.7)	−0.6 (1.4)	0.677	48.8 (1.2)	50.0 (1.1)	0.4 (1.0)	0.695	0.541
RE	47.1 (1.8)	49.0 (1.6)	−0.2 (1.3)	0.885	49.5 (1.1)	49.9 (1.0)	−0.6 (0.9)	0.528	0.795
MH	48.8 (1.9)	50.0 (2.0)	−0.3 (1.2)	0.807	50.8 (1.2)	52.8 (1.1)	1.2 (0.9)	0.175	0.283
PCS	46.0 (1.3)	46.3 (1.6)	−0.1 (1.0)	0.961	47.3 (1.1)	49.3 (1.0)	2.0 (0.7)	0.004	0.074
MCS	48.9 (1.9)	50.5 (1.9)	0.2 (1.2)	0.892	50.4 (1.2)	51.9 (1.1)	0.7 (0.8)	0.427	0.704

Data given as unadjusted mean (SE) unless otherwise indicated. The analysis used an MMRM. The analyses included the fixed, categorical effects of rhPTH(1-84), treatment, region, sex, visit, and treatment-by-visit interaction, as well as the continuous, fixed covariates of age, baseline HRQoL score, and the baseline-by-visit interaction. An unstructured covariance structure shared across treatment groups was used to model the within-patient errors. For all domains, normal population has the mean of 50 and a standard deviation of 10. Abbreviation: RE, role-emotional.

^a^ LS mean change is the model-based mean change from baseline to week 24.

^b^ Within-treatment–group *P* value.

^c^ Between-treatment–group *P* value.

### Independent predictors or modifiers of change in HRQoL

#### Serum calcium

We found no significant correlation between baseline HRQoL and either serum calcium level at baseline or change in serum calcium level between enrollment and randomization (effect of optimization). Additionally, changes in HRQoL during the treatment phase were not correlated with changes in serum calcium level.

#### Baseline HRQoL

**Figure 1.**
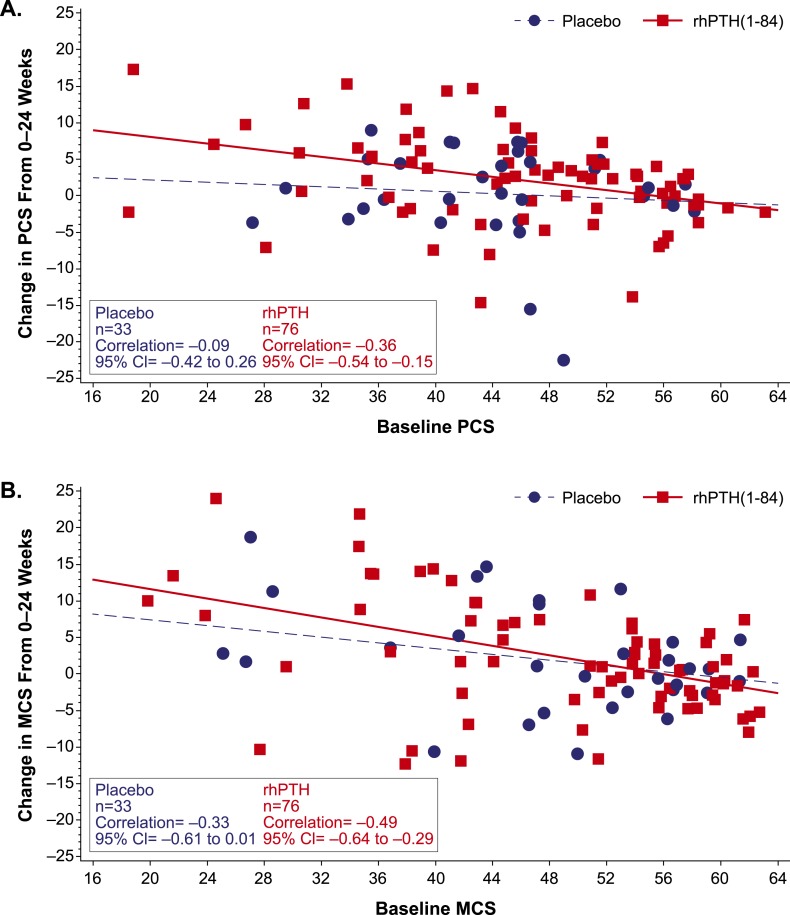
Change from baseline in SF-36 PCS and MCS scores. The change in (A) PCS score and (B) MCS score from baseline to week 24 is plotted as a function of baseline score in patients who received rhPTH(1-84) or placebo (red squares and blue circles, respectively). The regression line for rhPTH(1-84)-treated patients (solid red line) had a slope that did not encompass zero, whereas the slope of the line for placebo-treated patients (broken blue line) did. For all domains, normal population has the mean of 50 and a standard deviation of 10. CI, confidence interval.

For all SF-36 domains, baseline HRQoL score was a significant (negative) predictor of change in HRQoL after treatment (data not shown): patients who started with lower scores (*i.e.,* greater disability) had a greater increase from baseline. Figure 1 illustrates negative correlation between the baseline score and the magnitude of changes in PCS and MCS scores in response to treatment.

#### Sex

In this analysis, sex was included *a priori* in the MMRM analysis and appeared to confound the relationship between rhPTH(1-84) and HRQoL. We did not find a consistent effect of sex on change in HRQoL. When compared with men, women had an average 2.5 lower score for PF (*P* = 0.016), a 2.4 higher RP (*P* = 0.040), and a 3.1 higher score for role-emotional (*P* = 0.017). Additionally, the treatment effect of rhPTH(1-84), as measured by LS mean changes in HRQoL in the rhPTH(1-84) group, were substantially diminished for several domains (>10%) when sex was included in the model (RP by 35%, BP by 13%, MH by 31%, and the MCS score by 49%). Consequently, the magnitude and significance of improvement in HRQoL and the number of domains with significant improvement in the rhPTH(1-84)-treated group were diminished as a result of including sex in the model.

#### Thyroid function

At baseline, thyroid function test results were either normal or showed a suppressed but stable thyroid-stimulating hormone level (a common finding in patients with prior history of thyroid cancer). Furthermore, there were no changes in thyroid status between randomization and the end of study in either group (data not shown).

#### Geographic region

A preliminary MMRM analysis with an interaction term for treatment by region showed significance for several SF-36 domains indicating effect modification by region. Therefore, a sensitivity analysis was conducted to further examine the effect of rhPTH(1-84) on HRQoL when stratified by region.

The results show that the LS mean changes from baseline to week 24 in the SF-36 domains in patients from the sites within North America and Western Europe were similar to each other, whereas the SF-36 changes in the Hungarian patients were different. As shown in Table 4, rhPTH(1-84) treatment in the North American and Western European sites was associated with significant increases in change from baseline in seven of the 10 domains (PF, *P* < 0.001; RP, *P* < 0.05; BP, *P* < 0.05; GH, *P* < 0.05; VT, *P* < 0.001; MH, *P* < 0.05; PCS, *P* < 0.001), with no significant changes in the placebo group. In addition, comparison of the placebo and rhPTH(1-84) LS mean change from baseline values indicated significant differences between treatment groups for the RP (*P* < 0.05), VT (*P* < 0.05), and PCS (*P* = 0.01) domains. Conversely, at the Hungarian sites, there was no increase in scores from baseline in the rhPTH(1-84) treatment group but rather a trend toward decreased scores (not statistically significant).

**Table 4.** Regional SF-36 Scores at Baseline and After 24 Weeks of Treatment

SF-36 Domain	North America/Western Europe								
	Placebo (n = 32)				rhPTH(1-84) (n = 67)				rhPTH(1-84) vs Placebo
	Baseline	Week 24	LS Mean Change (SE)*^a^*	*P* Value*^b^*	Baseline	Week 24	LS Mean Change (SE)*^a^*	*P* Value*^b^*	*P* Value*^c^*
PF	46.4 (1.9)	46.8 (2.0)	0.9 (1.2)	0.461	49.2 (1.2)	51.4 (1.1)	3.0 (0.8)	<0.001	0.120
RP	47.8 (1.9)	47.5 (1.8)	−1.3 (1.3)	0.349	47.6 (1.2)	50.5 (1.1)	2.1 (0.9)	0.018	0.030
BP	48.1 (1.5)	47.7 (1.9)	−0.7 (1.5)	0.633	47.9 (1.3)	51.0 (1.3)	2.6 (1.0)	0.013	0.064
GH	47.7 (2.3)	48.6 (2.3)	0.5 (1.2)	0.675	46.3 (1.5)	49.0 (1.4)	2.1 (0.9)	0.016	0.282
VT	48.5 (2.1)	49.2 (2.2)	0.6 (1.4)	0.667	47.7 (1.5)	52.8 (1.2)	4.8 (1.0)	<0.001	0.013
SF	48.5 (1.8)	48.7 (1.9)	−0.4 (1.6)	0.784	48.2 (1.4)	50.2 (1.3)	1.2 (1.1)	0.255	0.364
RE	48.1 (2.0)	49.9 (1.7)	0.2 (1.3)	0.903	50.0 (1.2)	51.1 (1.0)	0.6 (0.9)	0.460	0.743
MH	49.5 (2.0)	50.6 (2.0)	−0.0 (1.3)	0.988	50.1 (1.4)	53.1 (1.2)	2.2 (0.9)	0.017	0.147
PCS	46.9 (1.6)	46.6 (2.0)	−0.5 (1.1)	0.640	47.2 (1.3)	50.0 (1.1)	2.7 (0.7)	<0.001	0.010
MCS	49.5 (2.1)	51.0 (1.9)	0.5 (1.3)	0.723	49.8 (1.4)	52.2 (1.2)	1.7 (0.9)	0.057	0.404

	Hungary								
	Placebo (n = 7)				rhPTH(1-84) (n = 16)				
PF	42.1 (1.7)	46.9 (2.3)	4.5 (3.3)	0.183	49.4 (1.9)	45.3 (2.6)	−2.8 (2.3)	0.244	0.065
RP	40.8 (2.4)	45.3 (4.1)	2.6 (3.1)	0.411	48.5 (2.5)	46.7 (2.4)	−2.6 (2.4)	0.290	0.157
BP	48.1 (4.1)	48.3 (4.0)	0.1 (4.3)	0.973	49.7 (3.0)	50.1 (3.4)	0.6 (3.3)	0.848	0.914
GH	41.1 (3.6)	39.1 (4.2)	−2.7 (2.7)	0.323	45.3 (2.7)	46.6 (2.6)	1.2 (2.0)	0.570	0.187
VT	45.0 (4.0)	53.0 (3.5)	6.4 (3.5)	0.088	55.9 (2.5)	53.8 (2.7)	−0.6 (2.6)	0.813	0.103
SF	46.6 (4.0)	48.0 (4.0)	1.1 (3.3)	0.752	51.1 (2.1)	48.9 (2.7)	−1.7 (2.4)	0.472	0.464
RE	43.2 (4.1)	45.7 (4.7)	−0.2 (3.6)	0.956	47.7 (3.0)	45.5 (2.9)	−4.0 (2.7)	0.151	0.345
MH	46.4 (5.1)	47.9 (5.9)	1.5 (2.2)	0.504	53.3 (2.3)	51.5 (2.6)	−2.2 (1.7)	0.223	0.148
PCS	42.5 (1.5)	45.2 (2.3)	2.6 (2.7)	0.345	47.8 (2.2)	46.7 (2.5)	−0.1 (2.1)	0.976	0.378
MCS	46.5 (5.1)	48.9 (5.5)	0.1 (3.1)	0.964	52.7 (2.4)	50.9 (2.7)	−4.1 (2.2)	0.085	0.254

Data given as unadjusted mean (SE) unless otherwise indicated. The analysis used an MMRM. The analyses included the fixed, categorical effects of rhPTH(1-84) treatment, sex, visit, and treatment-by-visit interaction, as well as the continuous, fixed covariates of age, baseline HRQoL score, and the baseline-by-visit interaction. An unstructured covariance structure shared across treatment groups was used to model the within-patient errors. For all domains, normal population has the mean of 50 and a standard deviation of 10. Abbreviation: RE, role-emotional.

^a^ LS mean change is the model-based mean change from baseline to week 24.

^b^ Within-treatment–group *P* value.

^c^ Between-treatment–group *P* value.

#### Temporal patterns in HRQoL

**Figure 2.**
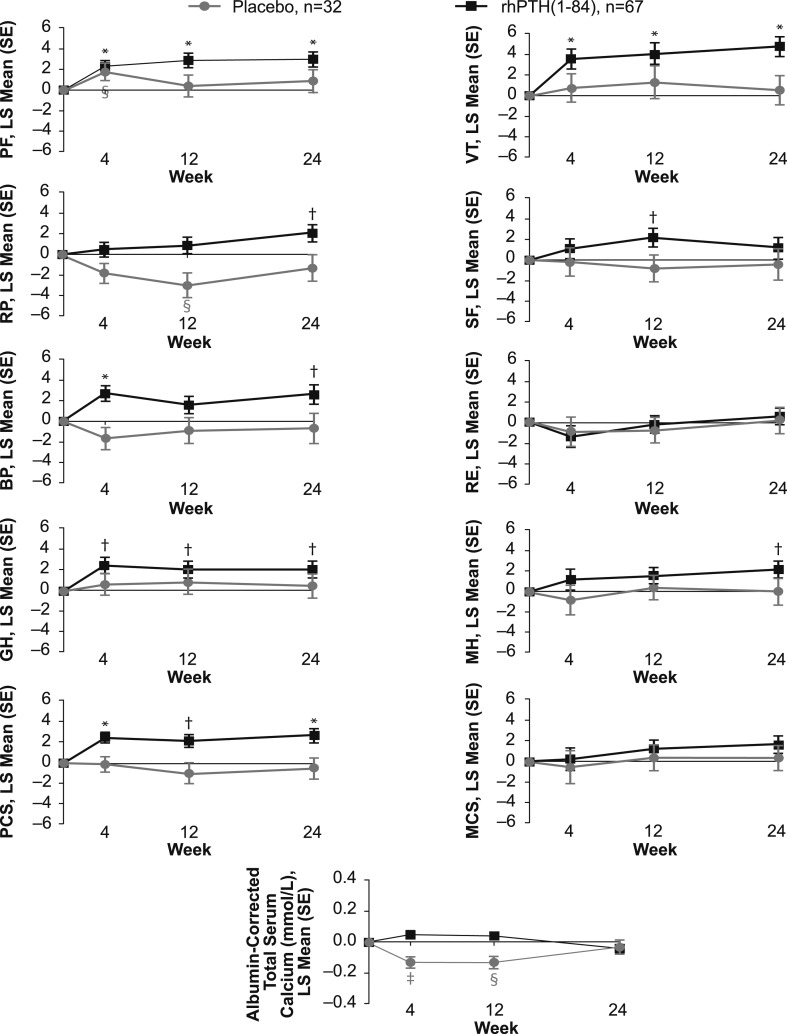
Change in SF-36 scores and albumin-corrected total serum calcium levels. A time-course plot of the change from baseline to weeks 4, 12, and 24 in patients from North American and Western European sites who received rhPTH(1-84) or placebo (black and gray symbols, respectively). *P < 0.001 for rhPTH(1-84) vs baseline; ^†^*P* < 0.05 for rhPTH(1-84) vs baseline; ^‡^*P* < 0.001 for placebo vs baseline; ^§^*P* < 0.05 for placebo vs baseline. RE, role-emotional; SE, standard error.

The statistically significant changes seen in HRQoL for the sites in North America and Western Europe were further investigated to more precisely delineate temporal changes throughout the time points at which the data were collected (*i.e.*, weeks 4, 12, and 24; Fig. 2). In patients who received rhPTH(1-84), increases in PF, GH, VT, and PCS were already observed at the earliest time point (week 4) and were maintained throughout the study. The increase in the BP domain observed at week 24 was also significant at week 4 but fluctuated midstudy. The RP and MH domains gradually increased and reached significance at week 24. In the placebo arm, HRQoL did not change for any domain (Fig. 2).

## Discussion

We examined HRQoL as measured by the SF-36 survey during the REPLACE clinical study, the pivotal trial that showed efficacy of rhPTH(1-84) for the treatment of adults with chronic hypoparathyroidism. The differences between the treatment groups were not statistically significant. In patients who received placebo, HRQoL did not change between randomization and 24 weeks of treatment despite achievement of target serum levels of calcium. In contrast, there were improvements in several SF-36 domains as well as the PCS score in patients treated with rhPTH(1-84). GH, VT, BP, and the PCS scores all had statistically significant increases from baseline in patients who received rhPTH(1-84). Given that the MID for the group mean for the individual domains of SF-36 version 2 and summary scores are between 2 and 3, we note that, on average, patients treated with rhPTH(1-84) in our study achieved the MID thresholds for the VT domain and for PCS when using the model-based mean change, and additionally for the BP domain when using the unadjusted mean change (Table 3). An important qualification to this and all studies of HRQoL in hypoparathyroidism is that the SF-36 is not a disease-specific instrument. As a generic health-status assessment questionnaire, it cannot assess the impact and symptoms specifically related to hypoparathyroidism. Although investigators await the development of a validated, disease-specific, patient-reported outcomes tool for hypoparathyroidism, the SF-36 is a standard, validated tool for measuring HRQoL in general. Although it has been shown that hypothyroidism is, itself, associated with reduced HRQoL (5), it is not likely that our results could be explained by abnormal thyroid status. Our patients had stable thyroid function test results at randomization and no change during the study.

Our results are consistent with findings from two previous open-label studies, one from New York (10, 11) and the other from Italy (12). However, the magnitude of increase in the HRQoL scores in the prior studies was greater than that observed in REPLACE (10–12), possibly because patients in REPLACE had higher baseline scores. It is important to note, however, that at least some HRQoL scores, although higher than in the aforementioned investigator-initiated studies, were nevertheless lower than those in the normal population, even after the optimization period that was part of the REPLACE design. It is unlikely that the differences in effect size among the three studies were due to the use of different versions of the SF-36 (version 1 in earlier open label studies; version 2 in REPLACE), because the two versions produce similar scores when used in the normal population (20). Another possible explanation is study design. Patients in REPLACE went through an optimization period of 2 to 16 weeks before randomization, during which supplement doses were adjusted and vitamin D deficiency and hypomagnesemia corrected. Albumin-corrected serum calcium levels increased by 0.46 mg/dL (0.12 mmol/L) during the optimization period. This improved biochemical control, in itself, may have improved the patients’ well-being, leading to the higher baseline scores, but we cannot prove this, because we did not have HRQoL data at screening. During optimization, patients had greater frequency of medical encounters and biochemical testing than is typical in clinical practice, and many had increases in serum calcium to levels that are higher than is usually recommended for this population. Despite that additional effort and biochemical improvement, some HRQoL scores still remained below normal after optimization of conventional therapy.

In contrast to the consistency of our results with the open-label studies mentioned, our findings from REPLACE are unlike those from, to our knowledge, the only other double-blind, placebo-controlled trial to use rhPTH(1-84) in patients with hypoparathyroidism. In the investigator-initiated study from Denmark (13), 62 patients were randomly assigned 1:1 to placebo or a fixed dose of rhPTH(1-84) 100 μg/d. Of note, doses of supplemental calcium or active vitamin D were not reduced unless a patient developed hypercalcemia, which occurred with higher frequency in patients receiving rhPTH(1-84). In response to treatment, scores improved in more domains in the placebo group than in the rhPTH(1-84) group, possibly because of higher prevalence of hypercalcemia in the latter. In contrast, our study shows improvements in several HRQoL domains in patients receiving rhPTH(1-84) and no improvements in patients receiving placebo, despite similar levels of serum calcium in the two groups at baseline and at 24 weeks. A possible limitation to our study is that the reductions in supplement doses may have led at least some of our patients to believe that they were randomly assigned to receive the active drug, which might have affected their responses to the SF-36 questionnaires. It is not likely, however, that the difference between our study and the Danish study could be attributed to patients in REPLACE guessing that they were receiving the active drug. Danish patients receiving the active drug also had dose reductions or elimination of supplement because they developed hypercalcemia with greater frequency than the placebo-treated patients (21).

Identifying patients who have the greatest potential to experience improvements in HRQoL with treatment is important because it could guide clinicians in selecting patients who should be considered for PTH therapy, in addition to the consideration of objective biochemical parameters. We sought potential predictors of HRQoL response by examining their associations with the changes in the SF-36 scores using the REPLACE trial data. In a separate analysis, we found that routinely measured fasting serum calcium levels at baseline or at 24 weeks, or the differences between the two, were not associated with the HRQoL responses. This observation does not necessarily indicate that control of serum calcium has no bearing on HRQoL. One way in which clinical data could fail to detect a relationship between serum calcium level and HRQoL is that single calcium measurements may not reflect serum calcium levels throughout the entire day, which may have a greater influence on overall well-being. The mechanism by which rhPTH(1-84) treatment improves HRQoL could not be elucidated in this study but may be related to more stable (or less variable) 24-hour profile of serum calcium level (which could not be assessed in the study), or an as yet unidentified effect of rhPTH(1-84).

Interestingly, we found that baseline HRQoL scores negatively correlated with response such that patients with low baseline scores (*i.e.,* greater disability) had greater improvements in scores. This suggests that patients with poor HRQoL while receiving conventional therapy might receive this added benefit from rhPTH(1-84) treatment. Although sex was a significant confounder in the analysis, there were not consistent sex-based differences in the response, suggesting that women and men may experience a HRQoL benefit from PTH therapy.

Because REPLACE was an international study, it afforded the opportunity to examine whether the effects are influenced by geographic region. We observed differences in baseline HRQoL scores, with the highest values reported by patients from North America and lower values reported by patients in sites in Western Europe and Hungary. In addition, at Hungarian sites (Table 2), baseline HRQoL scores were not balanced between treatment arms, with a tendency toward higher scores in patients randomly assigned to receive rhPTH(1-84). Because we have observed that higher baseline scores were a negative predictor of response to rhPTH(1-84) in the entire study population, this imbalance may have contributed to the finding that patients from Hungarian sites did not show improvement with rhPTH(1-84) treatment. In addition, the investigator from Hungary (P. Lakatos, personal communication) has suggested that there may have been a misinterpretation of the questionnaires by their patients despite their use of a validated translation into Hungarian. Our data do not allow us to determine the basis of these differing findings at Hungarian sites. Of note, in the other two regions (North America and Western Europe), which had balanced baseline scores between the groups, there was a significant increase in several scores in rhPTH(1-84)-treated but not in placebo-treated patients, with the differences between the groups reaching statistical significance for at least some domains (Table 4).

We also examined the time course of HRQoL changes during REPLACE. For the domains that showed the greatest improvements in the rhPTH(1-84)-treated patients, the significant changes were already noted at 4 weeks and persisted through the study (Fig. 2), which is consistent with data observed in the open-label study from New York (10). It is not likely that these improvements were simply due to the changes in serum calcium level, because we found no significant correlation between serum calcium level and changes in the SF-36 scores. Furthermore, the time course of SF-36 changes does not parallel the changes in serum calcium level seen in Fig. 2. It should also be noted that at 24 weeks, patients in the rhPTH(1-84) group had higher SF-36 scores compared with placebo-treated patients, despite the serum calcium level being practically identical in the two groups (Fig. 2).

In conclusion, we found that although there was no overall between-group difference, HRQoL improved in several domains in the rhPTH(1-84)-treated group but not in the placebo-treated group. Furthermore, in patients from North America and Western Europe, the HRQoL improvements in response to rhPTH(1-84) were greater and were statistically significantly different from placebo. The greatest improvements were noted in those who started with the lowest HRQoL levels. Thus, poor HRQoL in patients receiving conventional therapy may represent a clinical situation in which a trial of rhPTH(1-84) therapy could be considered.

## Abbreviations:

BP: bodily pain
GH: general health
HRQoL: health-related quality of life
LS: least squares
MCS: mental health component summary
MH: mental health
MID: minimally important difference
MMRM: mixed-effect model for repeated measures
PCS: physical component summary
PF: physical functioning
PTH: parathyroid hormone
rhPTH(1-84): recombinant human parathyroid hormone (1-84)
SF: social functioning
SF-36: 36-Item Short-Form Health Survey
VT: vitality.
